# Impact of laparoscopy on the prevention of pulmonary complications after thoracoscopic esophagectomy using data from JCOG0502: a prospective multicenter study

**DOI:** 10.1007/s00464-017-5716-5

**Published:** 2017-08-04

**Authors:** Isao Nozaki, Junki Mizusawa, Ken Kato, Hiroyasu Igaki, Yoshinori Ito, Hiroyuki Daiko, Masahiko Yano, Harushi Udagawa, Satoru Nakagawa, Masakazu Takagi, Yuko Kitagawa

**Affiliations:** 1 Japan Esophageal Oncology Group of Japan Clinical Oncology Group (JCOG), Tokyo, Japan; 2 Department of Surgery, Shikoku Cancer Center Hospital, 160 Minami-umemoto, Matsuyama, 791-0280 Japan; 3 Japan Clinical Oncology Group Data Center, National Cancer Center, Tokyo, Japan; 4 Gastrointestinal Medical Oncology Division, National Cancer Center Hospital, Tokyo, Japan; 5 Esophageal Surgery Division, National Cancer Center Hospital, Tokyo, Japan; 6 Department of Radiation Oncology, National Cancer Center Hospital, Tokyo, Japan; 7 Esophageal Surgery Division, National Cancer Center Hospital East, Kashiwa, Japan; 8 Department of Surgery, Osaka Medical Center for Cancer and Cardiovascular Diseases, Osaka, Japan; 9 Department of Gastroenterological Surgery, Toranomon Hospital, Tokyo, Japan; 10 Department of Surgery, Niigata Cancer Center Hospital, Niigata, Japan; 11 Department of Surgery, Shizuoka General Hospital, Shizuoka, Japan; 12 Department of Surgery, Keio University School of Medicine, Tokyo, Japan

**Keywords:** Minimally invasive esophagectomy, Thoracoscopy, Laparoscopy, Pneumonia, Esophageal cancer

## Abstract

**Background:**

Postoperative pulmonary complications (PPCs) are the most common causes of serious morbidity after esophagectomy, which involves both thoracic and abdominal incisions. Although the thoracoscopic approach decreases PPC frequency after esophagectomy, it remains unclear whether the frequency is further decreased by combining it with laparoscopic gastric mobilization. This study aimed to determine the impact of laparoscopy on the prevention of PPCs after thoracoscopic esophagectomy using data from the Japan Clinical Oncology Group Study 0502 (JCOG0502).

**Methods:**

JCOG0502 is a four-arm prospective study comparing esophagectomy with definitive chemo-radiotherapy. The use of thoracoscopy and/or laparoscopy was decided at the surgeon’s discretion. PPCs were defined as one or more of the following postoperative morbidities grade ≥2 (as per Common Terminology Criteria for Adverse Events v3.0): pneumonia, atelectasis, and acute respiratory distress syndrome.

**Results:**

A total of 379 patients were enrolled in JCOG0502. Of these, 210 patients underwent esophagectomy via thoracotomy with laparotomy (*n* = 102), thoracotomy with laparoscopy (*n* = 7), thoracoscopy with laparotomy (*n* = 43), and thoracoscopy with laparoscopy (*n* = 58). PPC frequency was reduced to a greater extent by thoracoscopy than by thoracotomy (thoracoscopy 15.8%, thoracotomy 30.3%; *p* = 0.015). However, following thoracoscopic esophagectomy, laparoscopy failed to further decrease the PPC frequency compared with laparotomy (laparoscopy 15.5%, laparotomy 16.3%; *p* = 1.00). Univariable analysis showed that thoracoscopy (shown above) and less blood loss (<350 mL 16.3%, ≥350 mL 30.2%; *p* = 0.022) were associated with PPC prevention, whereas laparoscopy showed a borderline significant association (laparoscopy 15.4%, laparotomy 26.9%; *p* = 0.079). Multivariable analysis also showed that thoracoscopy and less blood loss were associated with PPC prevention.

**Conclusion:**

Thoracoscopic approach to esophagectomy significantly reduced PPC frequency with minimal additional effect from laparoscopic gastric mobilization.

Esophageal cancer is one of the most aggressive cancers affecting the gastrointestinal tract and is known to have a poor outcome. Approximately 482,300 new cases and 406,800 deaths from esophageal cancer occurred worldwide in 2008 [1]. In Japan, these numbers were 20,556 and 11,592, respectively [2].

Esophagectomy is the standard treatment for potentially resectable thoracic esophageal cancer [3]. It typically consists of transthoracic esophageal resection and transabdominal gastric mobilization for esophageal replacement. However, because it includes a wide surgical excision, esophagectomy is associated with a higher risk of postoperative morbidity and mortality than those in other cancer surgeries [4, 5]. Postoperative pulmonary complications (PPCs) are the most common causes of serious morbidity after esophagectomy and can result in a poor prognosis in esophagectomized patients [6–8]. Therefore, PPC prevention is crucial to improve the survival of patients with esophageal cancer.

Thoracoscopic esophagectomy was first introduced in 1992 [9] and has been extensively performed in recent years [10]. This approach minimizes the extent of chest trauma. Several studies, including those from our group, have reported that it decreases PPC frequency [11–14]. However, it remains unclear whether PPC frequency is further decreased by combining this thoracoscopic esophagectomy with laparoscopic gastric mobilization because previous prospective studies have only evaluated the combined impact of the two approaches [13, 14]. The present study uses data from our prospective multicenter trial, the Japan Clinical Oncology Group Study 0502 (JCOG0502), to determine the effect of laparoscopy on the prevention of PPCs after thoracoscopic esophagectomy and the factors playing a role in this prevention.

## Materials and methods

### Study design and patient selection

**Fig. 1:**
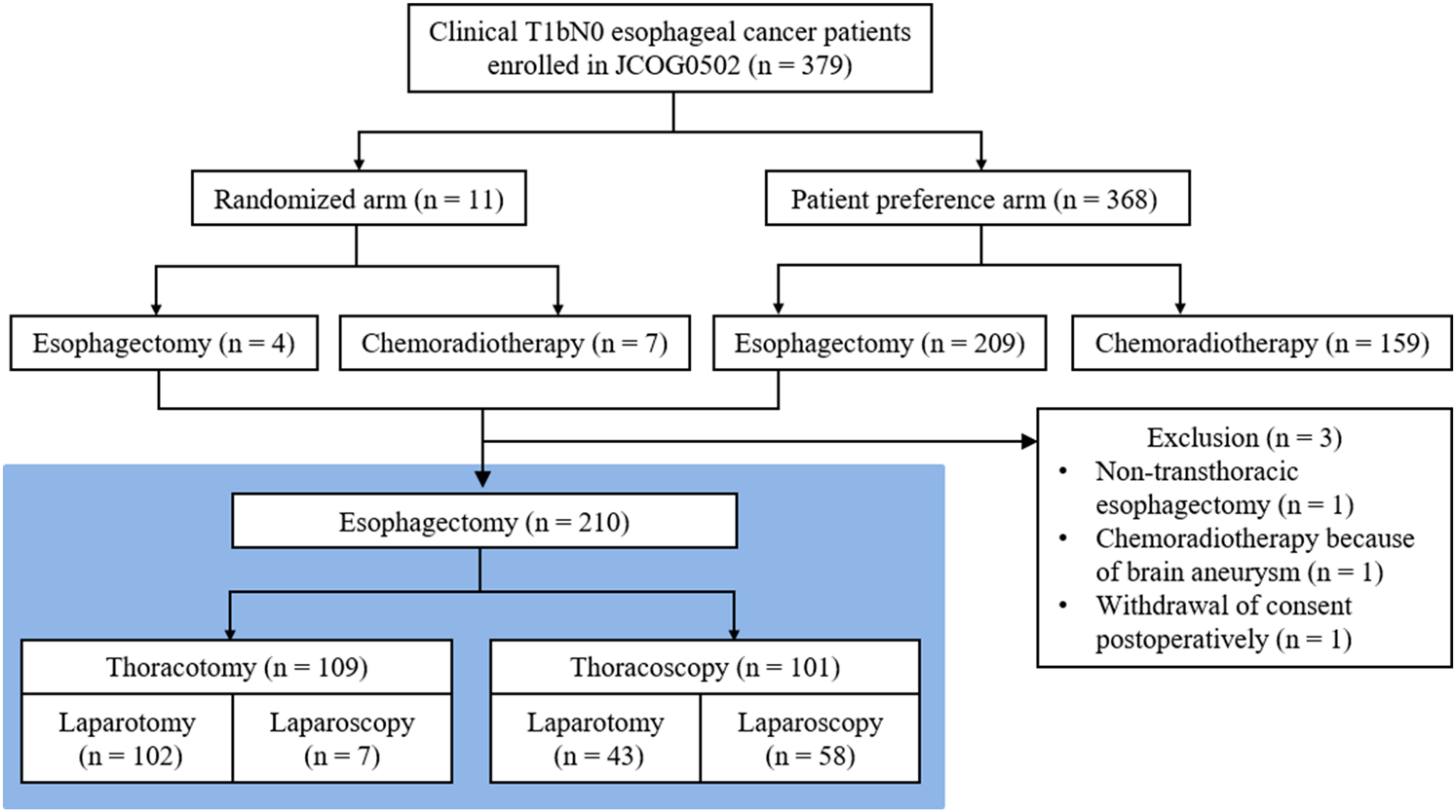
Flow diagram for the Japan Clinical Oncology Group (JCOG) 0502 study, with the present study highlighted in *light blue* (Color figure online)

JCOG0502 is a four-arm prospective study comparing esophagectomy with definitive chemo-radiotherapy for T1bN0 cancers and includes randomized and patient preference arms. Patients were assigned for randomization if they had no strong preference and were then randomly allocated to one of the two treatments (Fig. 1). However, if patients had a strong preference and refused randomization, they were allocated to the arm with their preferred treatment. Written informed consent was obtained from all enrolled patients. The study protocol was approved by the Clinical Trial Review Committee of the JCOG and by the review boards of all the participating institutions. The trial was registered with UMIN-CTR (www.umin.ac.jp/ctr/) (registration number: UMIN000000551). Patient accrual for this study has been completed. The primary endpoint is overall survival in the randomized arm, which will be analyzed in 2018. The key eligibility criteria for JCOG0502 were as follows: age between 20 and 75 years, diagnosis of histologically proven clinical stage IA (T1bN0) squamous cell carcinoma, adenosquamous cell carcinoma, or basaloid cell carcinoma in the thoracic esophagus according to the American Joint Committee on Cancer Staging Manual (7th edition), and performance status 0–1 according to the Eastern Cooperative Oncology Group. The major exclusion criteria were as follows: double primary cancer, uncontrolled diabetes, recent myocardial infarction (≤3 months), unstable angina, chronic obstructive pulmonary disease, pulmonary fibrosis, and heart failure.

### Operative methods

After the patients were allocated to the surgery arms, subtotal esophagectomy with lymphadenectomy was performed without preoperative chemotherapy and/or radiotherapy. The use of thoracoscopy and/or laparoscopy was decided at the surgeon’s discretion without any intention to compare these approaches, regardless of randomization or patient preference. Thoracoscopic esophagectomy was performed in the prone or lateral decubitus position according to the standards of each participating institution. In case of laparoscopic surgery, a minilaparotomy was made through which gastric conduits were created extracorporeally and specimens were removed. It could also be used as a hand-access port. Patients with upper thoracic disease underwent three-field lymphadenectomy, whereas those with mid- or lower thoracic disease underwent either two- or three-field lymphadenectomy at the surgeon’s discretion.

### Definitions and statistical methods

PPCs were defined as one or more of the following postoperative morbidities grade ≥2 as per Common Terminology Criteria for Adverse Events (CTCAE) v3.0: pneumonia, atelectasis, and acute respiratory distress syndrome. Other postoperative complications were defined as adverse events of grade ≥2 as per CTCAE v3.0. Postoperative mortality was defined as postoperative death within 30 days due to any cause, or death during the same hospital admission. Preoperative laboratory data were divided into two categories based on the median values. To compare open and thoracoscopic/laparoscopic surgery groups, the Wilcoxon rank-sum test and Fisher’s exact test were used for continuous data and categorical data, respectively. Multivariable analysis included logistic regression without variable selection as well as stepwise variable selection (with *α* = 0.2 for including and/or retaining a variable in the model). Baseline characteristics, preoperative laboratory data, and operative factors were included as explanatory variables in the multivariable analyses. The level of significance was set at a two-sided *p* value of <0.05. All analyses were performed using SAS software, v9.2 (SAS Institute Inc., Cary, NC) at the JCOG Data Center. The data presented in this article include those up to June 2015.

## Results

A total of 379 patients with clinical stage IA (T1bN0) thoracic esophageal cancer from 37 institutions were enrolled in JCOG0502 between December 2006 and February 2013. Excluding one patient who withdrew consent postoperatively, 210 of 379 patients underwent transthoracic esophagectomy (Fig. 1). These patients were enrolled from 30 institutions, and the median number of patients from each institution was 5 (range, 1–28). Gastric pull-up reconstruction was performed in the majority of patients (*n* = 206) with the colon being used as a conduit in the remaining patients (*n* = 4). Cervical anastomosis was performed in 198 patients, whereas intrathoracic anastomosis was performed in 12 patients. Of the 210 patients, 102 underwent thoracotomic esophagectomy with laparotomy, 7 underwent thoracotomy with laparoscopy, 43 underwent thoracoscopy with laparotomy, and 58 underwent thoracoscopy with laparoscopy (Fig. 1).

### Impact of thoracoscopy on PPC prevention

First, we compared thoracotomy (*n* = 109) and thoracoscopy (*n* = 101) to confirm the effect of thoracoscopic approach on PPC prevention. As shown in Table 1, there were no significant differences in the baseline characteristics of patients between the two approaches. Operative data showed that the thoracoscopic approach reduced blood loss but prolonged the operating time. Postoperatively, patients undergoing thoracoscopy had a lower PPC frequency than those undergoing thoracotomy [thoracoscopy 16 (15.8%), thoracotomy 33 (30.3%); *p* = 0.015], particularly atelectasis [thoracoscopy 11 (10.9%), thoracotomy 24 (22.0%); *p* = 0.041] (Table 2). There were no significant differences in the incidences of other complications and postoperative stay duration between the two approaches.

**Table 1:** Baseline characteristics and operative factors

	A: Thoracotomy (*n* = 109)		B: Thoracoscopy (*n* = 101)		A vs B	C vs D
	Laparotomy (*n* = 102)	Laparoscopy (*n* = 7)	C: Laparotomy (*n* = 43)	D: Laparoscopy (*n* = 58)		
	*n* (%)	*n* (%)	*n* (%)	*n* (%)	*p* ^a^	*p* ^a^
Baseline characteristics						
Age (years)						
Median (range)	61.5 (41–75)	67 (58–72)	63 (52–75)	62.5 (48–75)	0.522	0.765
Gender						
Male	87 (85.3)	6 (85.7)	33 (76.7)	49 (84.5)	0.462^b^	0.441^b^
Female	15 (14.7)	1 (14.3)	10 (23.3)	9 (15.5)		
Performance status^c^						
0	102 (100)	7 (100)	43 (100)	57 (98.3)	0.481^b^	1.00^b^
1	0 (0)	0 (0)	0 (0)	1 (1.7)		
Body mass index						
Median (range)	22.4 (13.4–28.9)	20.2 (18.6–26.0)	22.9 (17.1–28.4)	22.5 (18.3–28.3)	0.934	0.718
Preoperative PaO_2_^d^						
Median (range)	87.7 (70.0–116.7)	89.6 (73.6–114.0)	86.0 (71.0–112.0)	88.2 (71.1–155.0)	0.963	0.163
Tumor location						
Upper thoracic	10 (9.8)	1 (14.3)	6 (14.0)	9 (15.5)	0.183^b^	1.00^b^
Mid-thoracic	63 (61.8)	3 (42.9)	29 (67.4)	38 (65.5)		
Lower thoracic	29 (28.4)	3 (42.9)	8 (18.6)	11 (19.0)		
Tumor size						
≤4 cm	72 (70.6)	4 (57.1)	29 (67.4)	41 (70.7)	1.00^b^	0.828^b^
>4 cm	30 (29.4)	3 (42.9)	14 (32.6)	17 (29.3)		
Operative factors						
Body position during thoracoscopy						
Prone	NA	NA	17 (39.5)	23 (39.7)	NA	0.990^b^
Lateral decubitus	NA	NA	26 (60.5)	35 (60.3)		
Lymphadenectomy						
Two-field	37 (36.3)	4 (57.1)	17 (39.5)	23 (39.7)	0.779^b^	1.00^b^
Three-field	65 (63.7)	3 (42.9)	26 (60.5)	35 (60.3)		
Lymph nodes harvested						
Median (range)	48 (19–120)	44 (28–54)	53 (21–120)	57 (18–120)	0.063	0.690
Blood loss (mL)						
Median (range)	448 (80–1833)	240 (45–350)	351 (0–4225)	283.5 (10–2020)	<0.001	0.282
Operating time (min)						
Median (range)	405 (222–638)	338 (280–420)	476 (310–791)	533.5 (355–871)	<0.001	0.014

*NA* not applicable

^a^Wilcoxon rank-sum test

^b^Fisher’s exact test

^c^Eastern Cooperative Oncology Group performance status

^d^Partial pressure of O_2_ in arterial blood

**Table 2:** Postoperative pulmonary complications and other outcomes

	A: Thoracotomy (*n* = 109)		B: Thoracoscopy (*n* = 101)		A vs B	C vs D
	Laparotomy (*n* = 102)	Laparoscopy (*n* = 7)	C: Laparotomy (*n* = 43)	D: Laparoscopy (*n* = 58)		
	*n* (%)	*n* (%)	*n* (%)	*n* (%)	*p* ^a^	*p* ^a^
Pulmonary complications (PPCs)	32 (31.4)	1 (14.3)	7 (16.3)	9 (15.5)	0.015	1.00
Pneumonia	17 (16.7)	1 (14.3)	2 (4.7)	6 (10.3)	0.063	0.461
Atelectasis	23 (22.5)	1 (14.3)	6 (14.0)	5 (8.6)	0.041	0.521
ARDS^b^	0 (0)	0 (0)	0 (0)	1 (1.7)	0.481	1.00
Recurrent nerve palsy	16 (15.7)	1 (14.3)	6 (14.0)	9 (15.5)	1.00	1.00
Anastomotic leak	14 (13.7)	1 (14.3)	2 (4.7)	5 (8.6)	0.120	0.696
Postoperative mortality	1 (1.0)	0 (0)	0 (0)	1 (1.7)	1.00	1.00
Postoperative stay duration (days)						
Median (range)	22 (10–162)	17 (17–32)	22 (9–114)	24 (12–185)	0.472^c^	0.514^c^

^a^Fisher’s exact test

^b^Acute respiratory distress syndrome

^c^Wilcoxon rank-sum test

### Impact of laparoscopy on PPC prevention

Next, we determined whether PPC frequency was further decreased when combined with laparoscopy. Among the 101 patients who underwent thoracoscopic esophagectomy, 43 underwent laparotomy and 58 underwent laparoscopy (Fig. 1). As shown in Table 1, there were no significant differences in the baseline characteristics of the patients between the two approaches. Operative data showed that laparoscopy prolonged the operating time. Postoperatively, there were no significant differences in PPC frequency [laparoscopy 9 (15.5%), laparotomy 7 (16.3%); *p* = 1.00] and other postoperative complications/outcomes between the two approaches (Table 2).

Among the 109 patients who underwent thoracotomic esophagectomy, PPCs occurred in 32 patients (31.4%) in the laparotomy group and in 1 patient (14.3%) in the laparoscopy group. There was no significant difference between the two groups (*p* = 0.673).

### Preventive factors for PPCs

Finally, we comprehensively analyzed the data from all 210 patients to determine if laparoscopy could aid in the prevention of PPCs. In univariable analysis, both thoracoscopy (shown above) and less blood loss (< 350 mL 16.3%, ≥350 mL 30.2%; *p* = 0.022) were associated with PPC prevention (Table 3), whereas laparoscopy showed a borderline significant reduction in PPC frequency (laparoscopy 15.4%, laparotomy 26.9%; *p* = 0.079). Multivariable analysis identified both thoracoscopy (Odds ratio, 0.40; 95% confidence interval, 0.16–1.04; *p* = 0.059) and preoperative partial pressure of O_2_ (PaO_2_) in arterial blood (Odds ratio, 0.50; 95% confidence interval, 0.24–1.02; *p* = 0.057) as preventive factors with borderline significance. Stepwise regression analysis showed that both thoracoscopy (Odds ratio, 0.46; 95% confidence interval, 0.23–0.92; *p* = 0.028) and less blood loss (Odds ratio, 0.50; 95% confidence interval, 0.25–0.99; *p* = 0.048) were associated with PPC prevention.

**Table 3:** Preventive factors for pulmonary complications

	Total	Univariable analysis		Multivariable analysis w/o variable selection		Multivariable analysis with stepwise selection	
	*n*	*n* (%)	*p* ^a^	OR (95% CI)^b^	*p* ^c^	OR (95% CI)^b^	*p* ^c^
Baseline characteristics							
Age (years)							
<65	129	29 (22.5)	0.739	1			
≥65	81	20 (24.7)		1.28 (0.60–2.71)	0.524		
Gender							
Female	35	9 (25.7)	0.669	1			
Male	175	40 (22.9)		0.75 (0.27–2.10)	0.586		
Body mass index							
<25	168	40 (23.8)	0.840	1			
≥25	42	9 (21.4)		0.63 (0.21–1.60)	0.334		
Tumor location							
Upper thoracic	26	6 (23.1)	0.970	1			
Mid-thoracic	133	32 (24.1)		0.99 (0.33–2.99)	0.985		
Lower thoracic	51	11 (21.6)		0.76 (0.20–2.91)	0.689		
Tumor size							
≤4 cm	146	31 (21.2)	0.291	1			
>4 cm	64	18 (28.1)		1.48 (0.70–3.13)	0.311		
Preoperative laboratory data							
WBC count							
<5800/μL	102	24 (23.5)	1.000	1			
≥5800/μL	108	25 (23.1)		1.09 (0.50–2.34)	0.836		
Hemoglobin							
<13.5/12.5^d^ g/dL	67	16 (23.9)	1.000	1			
≥13.5/12.5^d^ g/dL	143	33 (23.1)		0.78 (0.35–1.73)	0.538		
Platelet count							
<22.1 × 10^4^/μL	105	23 (21.9)	0.744	1			
≥22.1 × 10^4^/μL	105	26 (24.8)		1.25 (0.62–2.52)	0.539		
Total protein							
<7.0 g/dL	105	21 (20.0)	0.328	1			
≥7.0 g/dL	105	28 (26.7)		1.28 (0.85–3.70)	0.497		
Total bilirubin							
<0.7 mg/dL	104	19 (18.3)	0.103	1		1	
≥0.7 mg/dL	106	30 (28.3)		1.77 (0.85–3.70)	0.129	1.77 (0.90–3.52)	0.101
Serum creatinine							
<0.77 mg/dL	107	26 (24.3)	0.747	1			
≥0.77 mg/dL	103	23 (22.3)		0.86 (0.39–1.88)	0.703		
PaO_2_^e^							
<87.9 mmHg	105	29 (27.6)	0.192	1		1	
≥87.9 mmHg	105	20 (19.0)		0.50 (0.24–1.02)	0.057	0.53 (0.27–1.04)	0.065
Operative factors							
Thoracic approach							
Thoracotomy	109	33 (30.3)	0.015	1		1	
Thoracoscopy	101	16 (15.8)		0.40 (0.16–1.04)	0.059	0.46 (0.23–0.92)	0.028
Abdominal approach							
Laparotomy	145	39 (26.9)	0.079	1			
Laparoscopy	65	10 (15.4)		0.90 (0.33–2.44)	0.842		
Lymphadenectomy							
Two-field	81	19 (23.5)	1.000	1			
Three-field	129	30 (23.3)		0.94 (0.42–2.13)	0.887		
Blood loss							
<350 mL	104	17 (16.3)	0.022	1		1	
≥350 mL	106	32 (30.2)		1.27 (0.54–2.98)	0.587	2.01 (1.01–4.03)	0.048
Operating time							
<452 min	105	25 (23.8)	1.000	1			
≥452 min	105	24 (22.9)		2.00 (0.91–4.39)	0.083		

^a^Fisher’s exact test

^b^Odds ratio (95% confidence interval)

^c^Logistic regression model

^d^Cutoff value for male/female patients

^e^Partial pressure of O_2_ in arterial blood

## Discussion

The present study demonstrated that the laparoscopic approach had limited effect on PPC prevention after thoracoscopic esophagectomy. To the best of our knowledge, this is only the second study using data from a prospective multicenter trial that evaluated the effect of laparoscopy on PPC prevention after esophagectomy. The first phase III multicenter trial (MIRO trial) was conducted by Mariette et al. [15]. They reported that laparoscopy decreased PPC frequency to a greater extent than that by laparotomy under the condition of thoracotomy (laparoscopy 17.7%, laparotomy 30.1%; *p* = 0.037) [16]. This result is similar to that of the present study comparing laparoscopy with laparotomy following thoracotomic esophagectomy (laparoscopy 14.3%, laparotomy 31.4%), although the difference was not significant (*p* = 0.673) due to the lack of power. Therefore, our results are consistent with those of the MIRO trial, and laparoscopy is recommended to prevent PPCs under the condition of thoracotomy.

Another prospective randomized trial (TIME trial) reported that a combination of thoracoscopic and laparoscopic approaches decreased the frequency of pneumonia to a great extent compared with thoracotomy with laparotomy (thoracoscopy–laparoscopy: 9%, thoracotomy–laparotomy: 29%; *p* = 0.005) [13]. However, the extent to which each minimally invasive approach contributes to this reduction is still unclear. The present study demonstrated that laparoscopy showed a borderline significant reduction in PPC frequency, whereas thoracoscopy showed an independent and significant reduction in PPC frequency. Therefore, it is likely that the impact of thoracoscopy on the prevention of pneumonia in the TIME trial was more than that of laparoscopy.

It is well known that laparoscopic surgery maintains better postoperative respiratory function than open abdominal surgery [17, 18], thus potentially affecting PPC prevention after thoracoscopic esophagectomy. However, whether laparoscopy contributes to better postoperative respiratory function [19, 20] and reduction of PPC frequency [8, 19, 20] remains controversial. In the present study, laparoscopy failed to show any substantial effect on PPC prevention under this condition. We speculated that this is because the preventive effect of thoracoscopy was so dominant that it masked that of laparoscopy. Under the condition of thoracotomy, a laparoscopic approach could potentially have a substantial effect on PPC prevention.

Another possible explanation for laparoscopy failing to show a substantial preventive impact after thoracoscopic esophagectomy is the presence of a minilaparotomy. In the MIRO trial, surgeons created a gastric conduit intracorporeally using a pure laparoscopic approach without any minilaparotomy, and specimens were removed via the thoracotomic incision [15]. In contrast, gastric conduits in the laparoscopic group of the present study were created extracorporeally through a minilaparotomy, and specimens were removed via this incision. We speculate that the pain and discomfort caused by the minilaparotomy diminished the preventive effect of laparoscopy on PPCs. A laparoscopic approach without minilaparotomy may be required to further decrease PPC frequency after thoracoscopic esophagectomy.

The prone position with artificial pneumothorax is reported to have an advantage over the lateral decubitus position by avoiding total lung collapse, thereby decreasing the incidence of PPCs [21]. In the present study, the body positions were equally distributed between the laparotomy and laparoscopy groups as shown in Table 1. Therefore, it is not likely that the body position during thoracoscopic esophagectomy affected the results in the present study.

Multivariable analysis in the present study indicated that thoracoscopy and less blood loss were significant PPC preventive factors. It has previously been reported that less blood loss is associated with PPC prevention after esophagectomy [7, 22, 23]. Total blood loss during surgery is one of the parameters to estimate surgical stress in surgical risk scoring systems [24, 25], and the calculated risk score is well correlated with the postoperative morbidity and mortality rates after gastrointestinal surgery [26]. Therefore, it is likely that less blood loss is one of the PPC preventive factors after esophagectomy.

The median body mass index (BMI) in the present study (22.5) was lower than that in trials conducted in Western countries (24.0–25.0) [13, 16]. It has been reported that patients with a high BMI do not have increased risk of PPCs after esophagectomy compared with those with a normal BMI [27, 28]. Likewise, the present study showed no significant increase in PPC frequency in the BMI ≥25 group (Table 3). The pneumonia and PPC frequencies in the thoracotomy–laparotomy (control) group were reported to be 29 and 30.1% in the TIME and MIRO trials, respectively [13, 16], which are comparable with that of the present study (31.4%). Thus, it is not likely that the lower BMI affected the results in the present study.

The present study had some limitations. First, because it was designed as a non-randomized comparison with a limited number of patients, the results may have been affected by patient selection bias and low statistical power. Second, there are omitted preoperative patient variables of pulmonary function tests, smoking history, and comorbidities, which may influence PPC frequency. Third, esophagectomy was only performed for patients with stage IA esophageal cancers. Therefore, the results may not be generalized to advanced esophageal cancers, which require preoperative therapy and more invasive surgical manipulation. Finally, the results may have been influenced by different surgical techniques and perioperative patient care styles because they were carried out depending on the standards of each participating institution.

Our new randomized phase III trial, JCOG1409 (MONET trial), is currently underway to compare the efficacy and safety of thoracoscopic esophagectomy and thoracotomic esophagectomy. In the MONET trial, patients are further stratified based on whether they undergo laparotomy or laparoscopy for gastric mobilization, performed according to the standard of each participating institution [29]. We expect that the data from JCOG1409 will strengthen the conclusions of the present study.

In conclusion, the present study demonstrated that the thoracoscopic approach and less blood loss were significant factors in the prevention of PPCs after esophagectomy, whereas the laparoscopic approach had minimal effect on the prevention of PPCs after thoracoscopic esophagectomy.
